# A systematic review of the safety and efficacy on cognitive function of herbal and nutritional medicines in older adults with and without subjective cognitive impairment

**DOI:** 10.1186/s13643-023-02301-6

**Published:** 2023-08-17

**Authors:** Adele E. Cave, Dennis H. Chang, Gerald W. Münch, Genevieve Z. Steiner-Lim

**Affiliations:** 1 NICM Health Research Institute, Western Sydney University, Locked Bag 1797, Penrith, NSW 2751 Australia; 2 School of Medicine, Western Sydney University, Penrith, NSW 2751 Australia; 3 Translational Health Research Institute (THRI), Western Sydney University, Locked Bag 1797, Penrith, NSW 2751 Australia

**Keywords:** Cognition, Complementary medicine, Herbal medicine, Nutrition, Subjective cognitive impairment (SCI), Mild cognitive impairment (MCI), Dementia, Alzheimer’s disease, Systematic review

## Abstract

**Background:**

Subjective cognitive impairment (SCI) substantially increases dementia risk and is often conceptualised as the preclinical asymptomatic phase of the cognitive decline continuum. Due to the lack of pharmacological interventions available to treat SCI and reduce dementia risk, and the popularity of herbal and nutritional medicines, the primary aim of this review was to investigate the efficacy on cognitive function and safety of herbal and nutritional medicines (relative to a control) for older adults with and without SCI. The secondary aims were to describe the study characteristics and assess the methodological quality of included studies.

**Method:**

Five databases (Cochrane, MEDLINE, CINAHL, PsycInfo, and EMBASE) were searched from database inception with weekly alerts established until review finalisation on 18 September 2022. Articles were eligible if they included the following: study population of older adults with and without SCI, herbal and nutritional medicines as an intervention, evaluated cognitive outcomes and were randomised control trials.

**Results:**

Data were extracted from 21/7666 eligible full-text articles, and the risk of methodological bias was assessed (with SCI = 9/21; without SCI = 12/21). Most studies (20/21) employed parallel, randomised, placebo-controlled designs and were 12 weeks in length. Herbal supplements were widely used (17/21), namely a form of *Ginkgo biloba* (8/21) or *Bacopa monnieri* (6/21). Measures of cognition varied across studies, with 14/21 reporting improvements in at least one domain of cognitive functioning over time, in the intervention group (compared to control). A total of 14/21 studies were deemed as having an overall high methodological risk of bias, 6/21 had some concerns, and only one study (using an SCI population) was assessed as having a low risk of methodological bias.

**Conclusions:**

Overall, this review found that there is a low quality of evidence regarding the efficacy of cognitive function and safety of herbal and nutritional medicines for older adults with and without SCI, due to a high risk of bias across studies. Additionally, further work needs to be done in classifying and understanding SCI and selecting appropriate trial primary outcomes before future studies can more accurately determine the efficacy of interventions for this population.

## Introduction

Subjective cognitive impairment (SCI) is a self-perceived worsening of cognitive functioning, particularly in the area of memory, that cannot be verified by neuropsychological tests [1, 2]. SCI lies on a continuum of healthy cognitive ageing and is conceptualised as the preclinical phase of dementia (healthy cognitive ageing, to preclinical SCI, followed by prodromal mild cognitive impairment (MCI), then dementia) [2–4]. SCI is estimated to double the risk of future objective decline (MCI or dementia) [5, 6], carries an increased prevalence of Alzheimer’s disease biomarkers and impacts mental health (1 in 3 people) and functional ability (1 in 2 people) [7], making it an important area of focus for secondary prevention research and care.

It is estimated that the prevalence of SCI is 1 in 4 older adults aged 60 years and above, worldwide, with these numbers increasing rapidly each year [2]. Currently, there are no approved pharmacological interventions available, with many older adults experiencing SCI seeking alternative treatments [8]. Difficulty also lies with the assessment of SCI, as current diagnostic tools have been developed for MCI or dementia [8, 9]. Furthermore, inconsistencies in the categorisation of SCI (namely the division between healthy adults without SCI and those with SCI) are apparent in research [8, 9]. Due to the increased risk of dementia and high prevalence of SCI, high-quality research into effective treatments to improve cognitive functioning and prolong further decline is needed.

A review and meta-analysis conducted in 2018 investigated a variety of interventions (group psychological, cognitive, lifestyle and complementary and alternative medicines) for the treatment of SCI and their efficacy on psychological well-being, metacognition and objective cognitive performance [9]. The authors found that studies were generally of low quality; hence, no firm conclusions could be made about the efficacy of the interventions employed [9]. Whilst this review/meta-analysis is of great importance to furthering SCI treatment research, it did not explore the efficacy of single interventions on cognitive functioning, nor did they investigate this usage and efficacy in older adults without SCI.

Complementary medicines (CMs) are defined as a broad range of health care approaches that are not thought to be part of conventional medical care [10, 11]. CMs are classified into three primary categories of delivery: nutritional (e.g. herbs, dietary supplements), psychological (e.g. meditation, relaxation therapy) and physical (e.g. acupuncture, massage) [10]. CMs are becoming more widely available and used by older adults, particularly herbal and nutritional medicines for the treatment of chronic health conditions including, cardiovascular disease [12], diabetes [13] and dementia [14, 15]. Herbal medicines contain herbal substances or herbal preparations, with nutritional supplements/medicines containing vitamins, minerals and in combination formulas and herbal substances/preparations as well [11, 16]. The natural properties of these medicines make them attractive to individuals wanting to improve their general health and well-being [11].

The primary aim of this review was to investigate the efficacy of cognitive function and safety of herbal and nutritional medicines (compared to an appropriate control group) for older adults with and without SCI. The secondary aims were to describe the study characteristics and assess the methodological quality of included studies, utilising the Cochrane risk of methodological bias (ROB 2) tool. This is the first review, to our knowledge, that has investigated the use of herbal and nutritional medicines for older adults with and without SCI, in depth.

## Methods

This review is structured according to the Preferred Reporting Items for Systematic Reviews and Meta-Analyses (PRISMA) guidelines [17] and registered with the PROSPERO international database of systematic reviews on 7 May 2021 (#CRD42021244631). A protocol was not published for this review.

### Eligibility criteria

A scoping review was conducted in line with the study eligibility criteria which were determined as per the PICOS principles for systematic reviews [18]:

1. Population: older adults^1^ with and without subjective cognitive impairment (subjective cognitive impairment is a self-perceived worsening of cognitive functioning) [1, 2]
2. Intervention: herbal and nutritional medicines (herbal medicines containing herbal substances or herbal preparations, and/or nutritional supplements/medicines containing vitamins, minerals, fatty acids etc., separately or in combination formulas) [11, 16]
3. Comparisons: appropriate control group (non-active orally ingested placebo, orally ingested active control)
4. Outcome: measures of cognition (both standardised/validated and non-standardised/non-validated testing measures)
5. Study design: randomised control trials (parallel or cross-over)

The following are the inclusion criteria: chronic dosing studies over a period of 2 weeks or more, peer-reviewed articles fully accessible online and written in English that met the above PICOS criteria. The following are the exclusion criteria: reviews, case studies, editorials, conference proceedings, preclinical studies (both in vitro and in vivo), trial protocols, trial registrations, book chapters, abstracts only, peer-reviewed articles in which the study population had a diagnosis of mild cognitive impairment or dementia, did not include cognition as a primary or secondary endpoint, or employed a co-intervention such as cognitive training.

### Search strategy

Two researchers (AEC, GZS) reviewed the search strategy in consultation with an experienced librarian, prior to the commencement of scoping. Four databases were searched for peer-reviewed articles: Cochrane, MEDLINE, CINAHL and PsycInfo from inception to 4 August 2018, and a further fifth database, EMBASE, was searched on 14 September 2022. Weekly alerts were established across the five databases until review finalisation on 18 September 2022. A full list of keywords is detailed below in Table 1. The only modification to the search strategy was the exclusion of non-randomised controlled trials from the Cochrane database to reduce the number of records for screening. Reference lists of included studies were also searched to identify any further eligible studies. Studies that included multiple age groups were also included if they reported demographics and outcomes separately for older participants in line with the eligibility criteria.

**Table 1:** Keywords forming the search strategy of the review utilised for the five databases

Area and search number	Search terms
Subjective cognitive impairment (S1)	“subjective cognitive impairment” OR “SCI” OR “subjective cognitive complaint*” OR “SCC” OR “subjective memory complaint*” OR “SMC” OR “cognitive decline” OR “preclinical dementia” OR “preclinical Alzheimer*” OR “age associated cognitive decline” OR “age related cognitive decline” OR “age associated memory impairment”
Older adults without subjective cognitive impairment (S2)	“healthy ageing” OR “healthy aging” OR “older adult*”
Intervention (S3)	“herbal medicine” OR “Chinese medicine” OR “complementary medicine” OR “alternative medicine” OR “natural medicine” OR “vitamin* OR nutraceutical” OR “nutritional supplement” OR “Chinese herbal medicine” OR “traditional Chinese medicine” OR “ginkgo” OR “ginseng” OR “alpha-lipoic acid” OR “lipoic acid” OR “bacopa monnier*” OR “brahmi”
(S4)	S1 OR S2 AND S3

*indicates truncation

### Data extraction and appraisal

All titles and abstracts were first screened by one author (AEC) for inclusion or exclusion from the review. If there were uncertainties regarding suitability for inclusion, the second reviewer (GZS) would assist to collaboratively make a final decision. Full-text articles were reviewed by the two authors with disagreements of acceptability resolved by discussion. Study characteristics were then extracted for each full-text article. These characteristics included author(s) and study location, aim, study population (group, sex, mean age, standard deviation and range), diagnosis criteria/global cognition measure, study design and outcome measurement frequencies, intervention, dose and duration, measures of cognition and results (cognition, retention, adherence and adverse events).

A methodological risk of bias assessment was conducted in accordance with the Cochrane Review Process for Randomised Trials (ROB 2) [19]. The quality of trial design, conduct and reporting of the included studies was assessed. Separate risk of bias assessments was conducted for parallel [20–39] and cross-over design studies [40]. The risk of bias tool evaluates five domains: bias arising from the randomisation process, deviations from intended interventions (effect of assignment and adherence to intervention), bias due to missing outcome data and in the measurement of the outcome, and bias in the selection of the reported result [19]. The sixth domain of bias arising from period and carryover effects was also evaluated for the cross-over study [19]. One author (AEC) independently conducted the risk of bias assessment, with the second author (GZS), reviewing the outcomes.

Individual studies were assessed as *low risk*, *some concerns* regarding methodology and *high risk* based on each of the above-mentioned domains. Studies with one or more domains assessed as high risk or with some concerns for multiple domains were deemed overall as high risk. Those with at least one domain with some concerns were evaluated in this category. The risk of bias process was conducted to assess the methodological quality of studies in their published form; study authors were not contacted for further information. A qualitative synthesis approach to this review was taken due to the large variation of interventions and cognitive assessments utilised across the studies, for each of the populations.

## Results

### Study selection

**Fig. 1:**
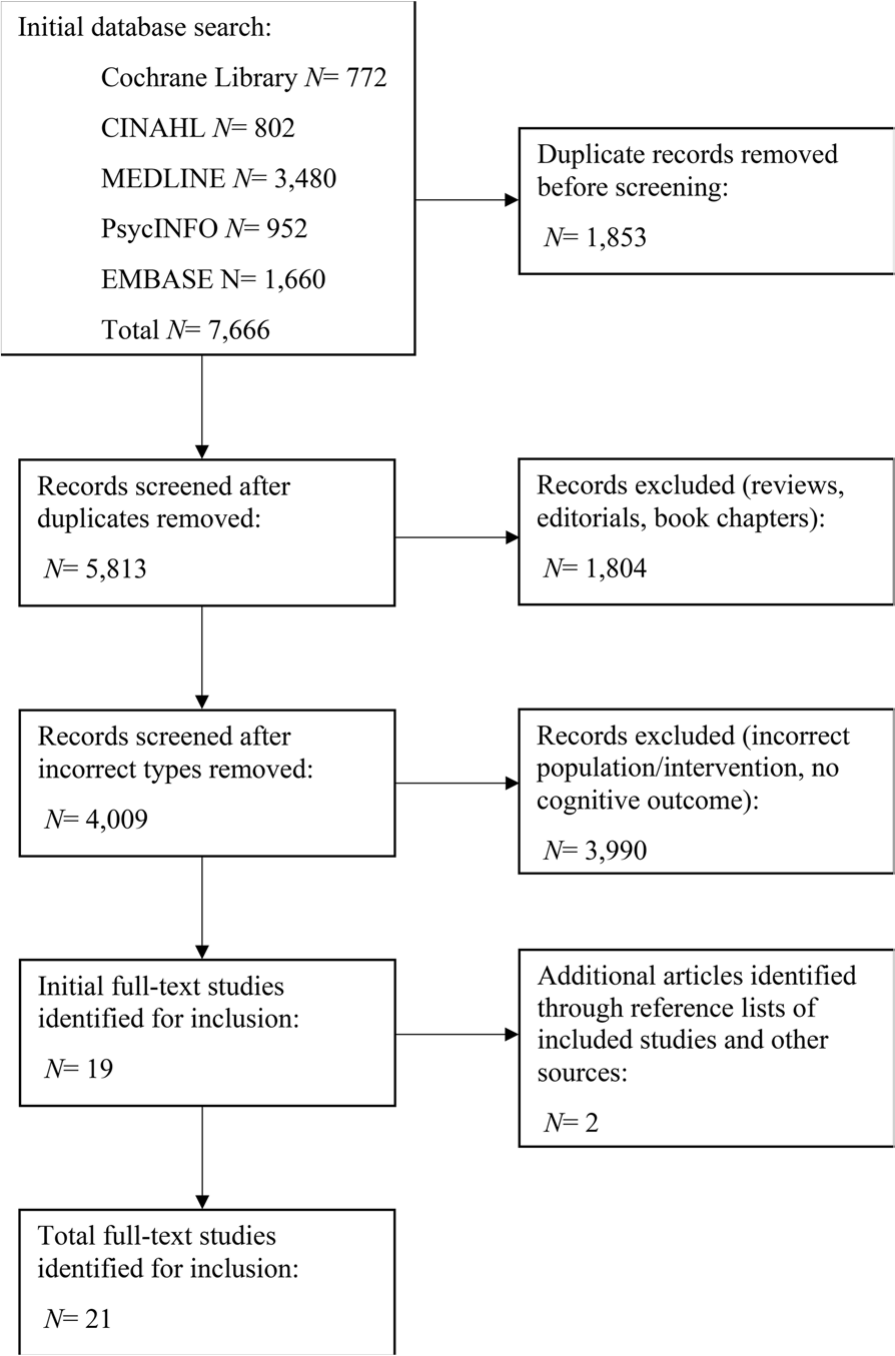
PRISMA flow diagram illustrating the study selection process

Figure 1 outlines the study selection process, with twenty-one studies meeting the eligibility criteria [20–40]. Nine studies involved older adults with SCI [20–22, 26, 28, 33, 35, 37, 38], and the remaining twelve, older adults without SCI [23–25, 27, 29–32, 34, 36, 39, 40].

### Study characteristics

Table 2 details a summary of the characteristics of the nine [20–22, 26, 28, 33, 35, 37, 38] SCI studies, and Table 3 details the twelve studies in older adults without SCI [23–25, 27, 29–32, 34, 36, 39, 40]. Both tables outline the study aim, population (group, sex, mean age, standard deviation and range), diagnosis/global cognition measure, design, intervention and dose, duration, measures of cognition and results (cognitive outcomes, retention and adherence, and adverse events).

**Table 2:** Summary of characteristics for studies involving older adults with subjective cognitive impairment

Author and study location	Aim	Study population	Diagnosis criteria/global cognition measure	Design	Intervention/control and dose	Duration	Measures of cognition	Results
Ban et al. 2018 *Korea* [20]	To examine the efficacy and safety of oral administration of *Tremella fuciformis* in individuals with subjective cognitive impairment	Community dwelling adults (65F:10 M) with subjective cognitive complaints aged *m* = 53.83 (± 5.5) years, range 40–65 years with 26F:4 M in the HD group and 26F:4 M in the LD group and 13F:2 M in the placebo group	MMSE (≥ 25), clinical dementia rating scale ≤ 0.5	Randomised, double-blind placebo control trial, outcomes at 0 and 8 weeks	*Tremella fuciformis* (species of fungus) 1) HD = 1200 mg daily (2 capsules, 3 × per day) 2) LD = 600 mg daily (2 capsules, 3 × per day) 3) Matched placebo (2 capsules, 3 × per day) N.R = placebo ingredients	8 weeks	Immediate recall from the pattern recognition memory task and the spatial span task (CANTAB), executive function (WCST)	Cognition: improvement in short-term memory and executive function in both HD and LD groups compared to placebo with a significant improvement in the HD group compared to placebo alone Retention/adherence: 69/75 participants completed the study: HD 93.3%, LD 90% and placebo 93.3%, with a > 90% adherence rate in all groups Adverse events: no serious AE’s were reported and no participants withdrew due to AE’s
Barbhaiya et al., 2008 *India* [21]	To evaluate the clinical efficacy, safety and tolerability of BacoMind® on impaired memory in elderly individuals	Adults with memory complaints for 1 year with no major cognitive deficits (AAMI) aged *m* = 64.98 (± 9.37) years, range 50–75 years with 23F:42 M	MMSE (≥ 24)	Randomised, double-blind placebo control trial, outcomes at 0, 12 and 24 weeks	BacoMind® (standardised extract of *Bacopa monnieri* with bioactive constituents) 1 × 450-mg capsule daily Matched placebo 1 × capsule daily, N.R = ingredients in placebo, only states ‘matched without actives’	12-week intervention period then a 12-week withdrawal period	Digit Span Forward and Backward (WAIS), Digit Cancellation Test, Serial Subtraction Test, list learning (RAVLT) immediate and delayed recall, passages (WMS-1) immediate and delayed recall, paired associates—similar and dissimilar pairs—immediate and delayed recall, visual retention 1 (based on designs), visual retention 2 (based on pictures), digit symbol (WAIS)	Cognition: significant group × time interaction (improvement) for intervention *c.f.* placebo in: digit span backward, list learning delayed recall, paired associates dissimilar delayed recall and visual retention Retention/adherence: 59/65 completed the study with 15 then excluded as outliers, participants taking ≥ 85% of medication were considered compliant Adverse events: no serious, mild or moderate adverse events reported by participants
Brautigam et al., 1998 *The Netherlands* [22]	To determine the efficacy and tolerance of two dosages of an alcohol/water extract of *Ginkgo biloba* versus placebo in the elderly with memory and/or concentration complaints	Elderly adults with self-reported memory and/or concentration complaints *m* = 68.96 (± 7.77) years, range 55–86 years with 31 males and 46 females in a high-dose group, 50F:32 M in a low-dose group and 47F:35 M in the placebo group	MMSE (≥ 20) and memory loss of known origin	Randomised, double-blind placebo control trial, outcomes at 0, 6, 12 and 24 weeks	*Ginkgo biloba* alcohol/water extract (composition: *Ginkgo biloba* leaf, folium, ethanol, flavone glycosides) 1) High dose (40 drops, 1.9 ml undiluted 3 × daily) 2) Low dose (40 drops (1.9 ml extract with placebo 3 × daily) 3) Placebo (40 drops, 1.9 ml 3 × daily) (water-soluble chlorophyll powder dissolved in an alcohol/water mixture, (water insoluble chlorophyll was dissolved in alcohol) both solutions were mixed together, taste and colour of all 3 dosages similar	4 weeks washout then 24 weeks treatment	EMCT (measuring attention and concentration), Benton test of visual retention-revised (short-term visual memory), Rey test parts 1 and 2 (short-term verbal memory and learning, long-term memory)	Cognition: no significant between-group improvements across time on the EMCT or Rey 1 and 2 tests. Within-group, Benton test scores increased in all 3 groups with the largest increase in the low-dose group Retention/adherence: total withdrawals from high dose group = 15, low dose = 14 and placebo = 15 (total retention rate of 81.7%) with an adherence rate of 93.98% Adverse events: 25 participants withdrew due to AEs including but not limited to gastro-intestinal complaints, dizziness, headache and sleepiness (across all groups)
Cicero et al., 2016 *Italy *[26]	To evaluate the effects of a rational assemblage of nutraceuticals on cognitive functions in a sample of 30 elderly subjects	30 older adults (*m* = 66 (± 3) years of age) (N.R = range, sex), with self-perceived cognitive decline	MMSE (20–27), self-perceived cognitive decline without a known diagnosis of cognitive decline or dementia	Randomised, double-blind placebo control trial, outcomes at 0 and 8 weeks	Combination of *Bacopa monnieri* dry extract 320 mg, l-Teanina 100 mg, *Crocus sativus* 30 mg, vitamin B6 9.5 mg, Biotine 450 mcg, folic acid 400 mcg, vitamin B12 33 mcg, vitamin D 25 mcg, copper 2 mg, capsule form and 1 per day Matched placebo, 1 × capsule per day, N.R = placebo ingredients	8 weeks	MMSE	Cognition: improvement in MMSE score in the active group Retention/adherence: no withdrawals from the trial, with 95% adherence in the active group and 90% in the placebo group Adverse events: one reported aftertaste after active product intake
Herrlinger et al., 2018 *USA* [28]	To investigate the effects of supplementation with a spearmint extract on cognitive performance, sleep and mood in individuals with AAMI	Adults with AAMI (based on the National Institute of Mental Health criteria) aged *m* = 59.4 (± 0.6) years, range 50–70 years with 30 participants in each group (placebo, 600 mg spearmint and 900 mg spearmint), (overall 30 M:60F)	MMSE (≥ 24), MAC-Q (≥ 25), WMS (≤ 29 on VPA-1) and WMS (≤ 9 on VPA-2)	Randomised, double-blind placebo control trial, outcomes at 0, 45 and 90 days (average on each day) (participants arrived on testing day fasting from product/food and were assessed 15 min prior to ingestion of intervention or placebo, then 30 min and 2, 4 and 6 h after ingestion. Average of scores across testing day equiv. to the overall score for that day)	1) 600 mg spearmint extract (*Mentha spicata* L.)/day (2 × capsules total) 2) 900 mg spearmint extract (*Mentha spicata* L.)/day (2 × capsules total) 3) Placebo capsules contained microcrystalline cellulose/day (2 × capsules total), N.R = if matched on taste, appearance and smell	90 days	CDR − with 11 tasks investigating attention and information processing, episodic and working memory, executive function and tracking (motor control)	Cognition: 900 mg spearmint group significantly improved in working memory and spatial working memory accuracy Retention/adherence: 3 participants withdrew (1 from the placebo group and 2 from the 600 mg group due to adverse events with a 98.1% (placebo), 99.1% (600 mg) and 100% (900 mg) adherence Adverse events: three participants reported AE’s: knee pain, myalgia, headache, worsening of oily scalp, cystic acne and heartburn. All deemed ‘not related’ with the exception of heartburn in both the placebo and 600 mg intervention groups
Macpherson et al., 2012 *Australia* [38]	To investigate the effects of 16-week supplementation of a multivitamin and herbal formula on cognition in community-dwelling, elderly women	Women reporting subjective memory complaints multivitamin group (*m* = 71.9 (± 4.8) years, placebo group (*m* = 70.3 (± 4.3) years, range 64–82 years with 28 participants in each group	MMSE (≥ 24), memory questionnaire to screen for SMCs and medical exam with a medical practitioner to determine whether participants were in good health	Randomised, double-blind placebo control trial, outcomes at 0 and 16 weeks	Swisse Women’s Ultivite 50 + ™ tablet (combination formula with 46 ingredients, with the largest quantities being from *Silybum marianum* dry fruit (St. Mary’s thistle), *Ginkgo biloba* and *Vitis vinifera* dry seed (grape seed), 1 tablet each day with breakfast. Placebo tablet matched on appearance of intervention, contained starch and 2 mg of riboflavin (vitamin B2), 1 tablet taken with breakfast	16 weeks	Swinburne University computerised cognitive assessment battery (SUCCAB) and CVLT-2 (word list learning)	Cognition: spatial working memory reaction time improved significantly for the intervention group, across time, compared to placebo Retention/adherence: 51/56 completed the study. Adherence was an average of 97%, with participants < 80% excluded from the trial (only 1/56 non-compliant) Adverse events = 1 participant from intervention experienced nausea and vomiting, and one in placebo developed a mild rash, both participants withdrew from study
Neri et al., 1995 *Italy* [33]	To determine the influence of treatment (intervention) on two major domains of well-being and their interrelationships (psychological well-being—affection and cognitive functioning and perceived quality of life)	Older adults (*m* = 60.45 (± 3.9) years, range 51–65 years) with AAMI, 21F:9 M in the intervention group and 21F:9 M in the placebo group	MMSE (≥ 24)	Randomised, double-blind placebo control trial, outcomes at 15 days, 3, 6 and 9 months (only first and last session analyses conducted)	Standardised ginseng extract G115 (N.R = dose) (from the root of *Panax ginseng*), dimethylaminoethanol bitartrate, minerals and vitamins (not specified)—two capsules daily (one after breakfast, one after lunch). Placebo ‘identical’ to study drug—two capsules daily (one after breakfast, one after lunch), N.R = placebo ingredients	9 months	RMT − (memory index, acquisition recall and deferred memory)	Cognition: memory index significantly increased in the treatment group compared to placebo at the endpoint Retention/adherence: no withdrawals reported in the study with a 92% adherence rate as an average across the duration of the study (reported at 3-month intervals) Adverse events: none reported by participants
Raghav et al., 2006 *India* [35]	To study the efficacy of SBME in subjects with AAMI	Forty adults with AAMI, range 55–70 years, N.R = mean age (± SD), (3F:37 M) with 20 participants in each group	MMSE (≤ 24) and WMS Logical Memory (< 6)^a^	Randomised, double-blind placebo control trial, outcomes at 0, 4, 8, 12 and 16 weeks	125 mg of SBME twice a day, N.R = type of administration. Placebo twice daily, N.R = administration, ingredients	12 weeks, then following 4 weeks of a placebo period	Mental control, logical memory, digit forward, digit backward, visual reproduction and paired associate learning (WMS)	Cognition: mental control (12 weeks), logical memory (4 weeks, 8 weeks and 12 weeks), digit forward (12 weeks) and paired associated learning (8 and 12 weeks) significantly improved at each of the time points listed during the intervention period Retention/adherence: 87.5% retention (2 participants in the placebo group and 1 in the SBME group withdrew), N.R = adherence Adverse events: 1 patient withdrew due to maculopapular rashes in the intervention group
Zhu et al., 2016 *China* [37]	To evaluate the safety and effectiveness of short-term administration of BrainPower Advanced, a multi-ingredient dietary supplement on SMCs in older adults	Community volunteers with SMCs aged *m* = 67.1 (± 10.5) years, range 47.28–88.43 years including 33F:14 M in the intervention group and 34F:17 M in the placebo group (ages and participant numbers calculated after withdrawals)	No reports of hypomnesis, forgetfulness, memory loss or impaired attention/concentration as determined by a standard medical questionnaire, constructed and validated for a Chinese population, participants scoring 1 (no SC or occasional slight SC) or 2 (slight/mild SC) were combined as ‘slight SC group’, 3 (moderate severe), 4 (severe) and 5 (very severe) were categorised as ‘severe SC group’, N.R = name of questionnaire	Randomised, double-blind placebo control trial, outcomes at 0 and 12 weeks	BrainPower Advanced (Phosphatidylserine, *Catharanthus roseus*, *Huperzia serrata* (whole plant), *Ginkgo biloba*, vitamin B6, vitamin B12, l-tyrosine, l-pyroglutamic acid, green tea extract (*Camellia sinensis* leaf), acetyl-l-carnitine, cola nut extract (*kola nitida*), choline bitartrate, l-glutamine, l-phenylalanine and l-cysteine) (2 capsules with meals daily Matched placebo, mainly containing flour, N.R = specific dose measurements for active and placebo (states 2 capsules with meals p/day) or additional placebo ingredients	12 weeks	Visual/auditory memory, abstracting and memory recall	Cognition: no significant between group improvements across time. Within group, both intervention and placebo outcomes improved across time—audio/visual memory, abstracting ability and memory retrieval (except memory retrieval for placebo) Retention/adherence: 98/101 participants completed the study, N.R = adherence Adverse events: 2 adverse event reports with withdrawal of participants due to gastrointestinal upset, N.R = which group they withdrew from

*AAMI* Age-associated memory impairment, *AE* Adverse event, *CANTAB* Cambridge Neuropsychological Test Automated Battery, *CDR* Cognitive drug research system, *CVLT-2* California Verbal Learning Task, *EMCT* Expended Mental Control Test, *HD* High dose, *LD* Low dose, *MAC-Q* Memory Complaint Questionnaire, *MMSE* Mini-Mental State Examination, *N.R* Not reported, *RAVLT* Rey Auditory Verbal Learning Test, *RMT* Randt Memory Test, *SBME* Standardised *Bacopa monnieri* extract, *SC* Symptom complaints, *SMCs* Subjective memory complaints, *VPA* Verbal paired associates, *WAIS* Wechsler Adult Intelligence Scale, *WCST* Wisconsin Card Sorting Test, *WMS* Weschler Memory Scale

^a^Raghav et al. (2006) [35] reported on AAMI without any evidence of dementia or psychiatric disorder despite excluding individuals scoring > 24 on the MMSE

**Table 3:** Summary of characteristics for studies involving older adults without subjective cognitive impairment

Author and study location	Aim	Study population	Diagnosis criteria/global cognition measure	Design	Intervention/control and dose	Duration	Measures of cognition	Results
Burns et al., 2006 *Australia* [23]	To extend on previous research in order to determine the efficacy of 120 mg ginkgo, to benefit performance of healthy older and young adults on cognitive outcomes	Healthy older adults *m* = 61.7 (± 5.5) range 55–79 years: 50 males (26 males in placebo, 24 in ginkgo) and 43 females (21 females in placebo, 22 in ginkgo) Younger adults also included in this study however analysed separately	Medical questionnaire—medical history and current medications, POMS, N.R = global cognition measure and names of medical questionnaires	Randomised, double-blind placebo control trial, outcomes at 0 and 12 weeks	Ginkgoforte™ (*Ginkgo biloba*) 120 mg per day (3 tablets daily of 40 mg each—1 per meal) Matched placebo (3 tablets per day—1 per meal), N.R = placebo ingredients	12 weeks	Woodcock-Johnson Psycho-educational battery-revised (fluid ability, crystallised ability, short-term memory, cognitive processing speed, long-term storage and retrieval, delayed recall, spot-the word (vocabulary), self-ordered pointing (executive function), odd-man-out (both movement and decision time), inspection time	Cognition: significant improvement in long-term storage and retrieval only in the ginkgo group Retention/adherence: 13 participants withdrew between pre- and post-treatment, adherence measured as ≥ 75% intake, all participants were compliant Adverse events: reported by 2 participants who withdrew in the ginkgo condition due to headaches and sleep disturbance
Calabrese et al., 2008 *U.S.A* [24]	To evaluate the effects of *Bacopa monnieri* (whole plant standardised dry extract) on cognitive function and its affect, safety and tolerability in healthy elderly participants	54 healthy older adults (60% women) living independently aged 65 years or older *m* = 73.5 years, N.R = range, S.D (27 participants in each group)	BOMC and no complaints of memory impairment, POMS, N.R = BOMC cut-off	Randomised, double-blind placebo control trial, outcomes at 0, 6 and 12 weeks	Standardised *Bacopa monnieri* extract, 300 mg/day (1 tablet daily) Matched placebo (1 tablet daily), N.R = placebo ingredients	6 weeks of placebo for control group and 12 weeks of *Bacopa monnieri* for the treatment group	Delayed recall score from RAVLT. Additionally, Stroop task assessing ability to ignore irrelevant information, the DAT and the WAIS letter digit test of immediate working memory	Cognition: significant improvement in RAVLT delayed recall and Stroop tasks for the *Bacopa monnieri* group Retention/adherence: 6 participants withdrew from study in total, average of 3.9 (out of 84) tablets missed with no difference between groups Adverse events: 10 in placebo group, 9 in the intervention group reporting flu like symptoms and digestive problems
Carlson et al., 2007 *USA* [25]	To determine if a *Ginkgo biloba*—containing supplement improves cognitive function and quality of life, alters primary haemostasis, and is safe in healthy, cognitively intact older adults	Healthy, cognitively intact older adults: ginkgo group (21F:21 M 73.1 (± 4.8) years range 65–84 years), placebo group (15F:21 M, 72.1 (± 6.0) years, range 65–83 years)	MMSE (24–29)	Randomised, double-blind placebo control trial, outcomes at 0 and 4 months	*Ginkgo biloba*-based supplement containing 160 mg of *Ginkgo biloba*, 68 mg of *Gotu kola*, 180 mg of DHA, a bioflavonoid concentrate (100 mg) and vitamin A (300 IU) as beta carotene, three capsules/day with meals. All participants were also given a once-daily multivitamin/multimineral supplement (Nutrilite Daily) Matched placebo three capsules/day with meals, N.R = placebo ingredients	4 months	Benton visual retention, controlled oral word association, judgement of line orientation, modified mini mental screening, list learning easy, list learning strict and symbol digit modalities	Cognition: significant improvement only in list learning strict task for the placebo group Retention/adherence: 3 participants from the Ginkgo group and 8 from the placebo group dropped out, > 95% adherence for all participants Adverse events: 17% of each group reported they had one AE attributed to the product they ingested, with the most common AEs being nausea/stomach upset and flatulence, two reports of headaches, rash and dizziness
Crews et al., 2005 *USA* [27]	To conduct the first known clinical trial of the short-term efficacy of cranberry juice on the neuropsychologic functioning of cognitively intact older adults	Cognitively intact older adults (> 60 years of age): cranberry group (25 adults) *m* = 69.17 (± 7.11) years placebo group (25 adults) *m* = 69.39 (± 5.80) (N.R = range, sex)	MMSE (≥ 24) and reporting no history dementia or significant neurocognitive impairment	Randomised, double-blind placebo control trial, outcomes at 0 and 6 weeks	32 oz/day (2 × 16 oz) of a beverage containing 27% cranberry juice sweetened with sucralose Matched placebo beverage 32 oz/day (2 × 16 oz), N.R = specific placebo ingredients	6 weeks	Selective Reminding Test, WMS-3 (face 1 and face 2 subtests), Trail Making Test (parts A and B), SCWT the WAIS-3 Digit Symbol-Coding subtest	Cognition: no significant improvements in neuropsychologic test data Retention/adherence: 3 participants excluded from analysis due to non-compliance, N.R = adherence Adverse events: no serious adverse effects were reported by participants, N.R = minor AEs
Crosta et al., 2020 *Italy* [39]	To evaluate the changes in the trail making test scores from baseline to 8 weeks of treatment with an antioxidant mix compared to placebo in healthy older adults	Healthy older adults (> 60 years of age): antioxidants mix group (28F:12 M, *m* = 61.88 (± 1.36) years), placebo group (27F:13 M, *m* = 62.05 (± 1.55) years) N.R = range	MMSE (≥ 27) and no neurological disorders	Randomised, double-blind placebo controlled trial, outcomes at 0 and 8 weeks	Antioxidant mix 1 tablet daily containing *Bacopa monnieri*, lycopene, astaxanthin and vitamin B12. Matched placebo tablet taken once daily containing magnesium vegetable stearate (5 mg) and non-active substances	1-week run-in period (unclear of what occurred during this time) followed by 8 weeks intake or intervention or placebo	TMT, VFT, MMSE, MoCA and RAVLT	Cognition: significant improvements in TMT A and TMT B, and VFT across time, and in the antioxidant group compared to placebo Retention/adherence: 2 participants excluded from the final analysis due to withdrawal (1 adverse event, 1 could not attend study sessions), compliance reported as 100% for nearly all participants Adverse events: 1 participant reported non-serious event of exacerbation of sinusitis in active group and 1 participant experienced an acute serious event of hepatitis E
Lewis et al., 2014 *USA* [29]	To extend the evaluative process of nutritional therapies through a randomised, double-blind, placebo-controlled clinical trial assessing a regimen of dietary supplements’ efficacy	Healthy older adults Ginkgo synergy® plus Choline *m* = 67.6 (± 6.3) years, range 58–82 years, with 25F:8 M; OPC Synergy® plus Catalyn group *m* = 68.5 (± 6.7) years, range 59–83 years, with 24F:7 M; placebo group *m* = 70.3 (± 8.3) years, range 60–93 years, with 21F:12 M	MMSE (≥ 23), SPMSQ, WMS-Story A, no AD or related disorders, not living in a nursing facility	Randomised, double-blind placebo control trial, outcomes at 0, 3 and 6 months	1) Ginkgo Synergy® (2 capsules/day—120 mg/day total) and choline (4 tablets/day to 700 mg/day total) 2) OPC Synergy® (2 capsules/day) and Catalyn (4 tablets/day) 3) Placebo containing cellulose, lactose and beet powder, N.R = placebo administration method/type, dose	6 months	MMSE, SCWT, the TMT Parts A and B, COWA, the Digit Symbol subtest of the WAIS and the HVLT-R	Cognition: according to time in the TMT-B the Ginkgo group showed improvement from baseline to 3 months, on the controlled oral word association trial-S scores significantly increased for the Ginkgo group from baseline to endpoint and in the OPC group from baseline to 3 months Retention/adherence: retention numbers inconsistently reported, N.R = adherence Adverse events: one participant in the OPC synergy group diagnosed with ulcerative colitis shortly after enrolling, one participant in the ginkgo synergy group reported joint aches and one placebo participant reported insomnia and heightened energy at night
Mix et al., 2000 *USA* [30]	To examine the relatively short-term (i.e. 6 weeks) efficacy of *Ginkgo biloba* extract EGB 761 on the cognitive functioning of cognitively intact persons over the age of 55 years via a diverse battery of neuropsychologic tests and measures	Cognitively intact healthy older adults, 55–86 years of age: ginkgo group *m* = 67.5 (± 9.23) years, placebo group *m* = 68.65 (± 6.95) years, 24F:24 M	MMSE (≥ 24) and no history of significant neurocognitive dysfunction, considered cognitively intact (self-reported)	Randomised, double-blind placebo control trial, outcomes at 0 and 6 weeks	*Ginkgo biloba* EGB 761 (180 mg/day = 60 mg × 3 capsules per day). Matched placebo × 3 capsules per day (methylcellulose), N.R = additional placebo ingredients	6 weeks	SCWT (parts A and B) and the WMS-revised, logical memory 1 and 2 and visual reproduction 1 and 2 subtests	Cognition: significant improvement in the intervention group across time in colour naming task within the Stroop Colour and Word test compared to placebo over the trial period Retention/adherence: 21 males and 19 females completed the study (40/44), 1 participant did not complete due to non-compliance (missing > 20% treatment), remaining 3/4 due to medical reasons, N.R = overall adherence Adverse events: no adverse effects reported by participants
Mix et al., 2002 *USA* [31]	To conduct the first known, large-scale clinical trial of the efficacy of *Ginkgo biloba* extract (EG 761) on the neuropsychological functioning of cognitively intact older adults	Cognitively intact older adults, ≥ 60 years of age: ginkgo group *m* = 66.97(± 6.12) years, placebo group *m* = 68.60 (± 6.96) years, (N.R = range), with 262 participants in total in the study, after withdrawals/non-compliance, 147F:102 M remained in the study	MMSE (≥ 26) and no history of dementia or significant neurocognitive impairment (self-reported)	Randomised, double-blind placebo control trial, outcomes at 0 and 6 weeks	*Ginkgo biloba* EGB 761 (180 mg/day = 60 mg × 3 tablets per day). Matched placebo tablets × 3 per day, N.R = placebo ingredients	6 weeks	SRT, WAIS-3 block design, Digit Symbol-Coding subtests, WMS-3 faces 1 and 2 subtests	Cognition: participants in the intervention group (compared to placebo) significantly improved in SRT tasks involving delayed free recall and recognition of visual material Retention/adherence: 249/262 participants completed the protocol, 5 from the placebo group and 4 from the intervention group were excluded from the study due to non-adherence (missing > 20% treatment), N.R = overall adherence Adverse events: total of 32 reported including but not limited to gastrointestinal, nervous system and respiratory/allergic reactions, across both groups. One serious event in the placebo group of an intracranial bleed
Morgan et al., 2010 *Australia* [32]	To investigate the effectiveness of *Bacopa monnieri* Linn. for improvement of memory performance in healthy older persons	Healthy older adults from the general population *m* = 65(± 7.53) years, range 55–86 years Bacopa group = 24F:25 M, placebo group = 28F:21 M	MMSE (≥ 24) and absence of depression (≤ 12) on HAMD	Randomised, double-blind placebo control trial, outcomes at 0 and 12 weeks	*Bacopa monnieri* (BacoMind™) 300 mg/day (one tablet daily). Matched placebo tablet, N.R = dose, ingredients	12 weeks	Audio-verbal and visual memory performance measured by RAVLT, the Rey-Osterrieth CFT and the Reitan TMT, subjective complaints measured by the MAC-Q	Cognition: *Bacopa* significantly improved verbal learning, memory acquisition and delayed recall, total learning and retroactive interference (RAVLT) Retention/adherence: 81/98 participants completed the study. N.R = adherence Adverse events: 9 in the treatment group, 2 placebo group (side effects occurred significantly more in the treatment group: increased stool frequency, nausea and abdominal cramps)
Perry et al., 2018 *UK* [34]	To evaluate for the first time the effects of a combination of sage, rosemary and Melissa—traditional European medicines on verbal recall in normal healthy subjects	Healthy older adults *m* = 61 (± 9.26) years (divided into 2 groups: younger (43–62), older (63–80)) 38F:6 M total (older group numbers: 4 males = 2 in the intervention group and 2 in the placebo group 14 females = 10 in intervention and 4 placebo)	No reported current or previous clinical diagnosis of cognitive impairment or dementia, N.R = global cognition measure	Randomised, double-blind placebo control trial, outcomes at 0 and 2 weeks	5 ml of SRM (*Salvia officinalis* L., *Rosmarinus officinalis* L. and *Melissa officinalis* L.) ethanol extract or 5 ml placebo (50% fresh sweet cicely (*Myrrhis odorata* (L.)), 1% Lyles Black Treacle and 1 g/ml 45% EtOH, diluted in water) twice per day, N.R = specific time of day for dose, or matched on smell, taste	2 weeks	Pen and paper immediate and delayed recall tests of verbal episodic memory	Cognition: no significant differences between the treatment or placebo groups from baseline to endpoint in immediate or delayed recall for the older group Retention/adherence: 1 participant did not complete the study (44/45 completed), an average missed dose of 2.2 for the older adults across both groups, N.R = specific adherence Adverse events: no adverse effects reported by participants
Solomon et al., 2002 *USA* [36]	To evaluate whether ginkgo improves memory in elderly adults as measured by objective neuropsychological tests and subjective ratings	Community-dwelling volunteer older adults (total of 132F:98 M, 115 in each group), aged between 60 and 82 years Ginkgo group *m* = 68.7 (± 4.7) years, with 65F:46 M, placebo group *m* = 69.9 (± 5.4) years with 63F:45 M (assignment to intervention numbers, F:M ratio and ages calculated after withdrawals)	MMSE (> 26) and in generally good health	Randomised, double-blind placebo control trial, outcomes at 0 and 6 weeks	Ginkgo (Ginkoba) 40 mg 1 tablet, taken 3 times per day. States matched placebo (lactose gelatin), 1 capsule 3 × per day. Note: active is a tablet, placebo is a capsule	6 weeks	Learning, memory, attention, concentration and expressive language tested utilising the CVLT, the Logical Memory subscale of the WMS-revised, the Visual Reproduction subscale, digit symbol subscale of the WAIS-revised), the digit span (WMS-R) and mental control (WMS-R) controlled category fluency test, Boston naming test	Cognition: no significant differences between the treatment groups on any outcome measure or between time points Retention/adherence: 4 ginkgo and 7 placebo withdrew consent with 88% of participants completing the trial, no significant differences between groups in non-completion, 7 participants in the ginkgo group and 9 in the placebo group did not comply with medication dosage, N.R = specific adherence Adverse events: not monitored in The study
Tohda et al., 2017 *Japan* [40]	Investigate the effects of a diosgenin-rich yam extract on cognitive enhancement in healthy volunteers	Healthy adults (16F:12 M) *m* = 46.5 (± 18.67) years, range 20–81 years (subgroup for analysis of older adults 60–81 years) (ages and participant numbers calculated after withdrawals)	Overall good physical and mental health, no diagnosis of AD or related disorders, N.R = global cognition measure	Randomised, double-blind placebo control, crossover trial, outcomes at 0, 12 and 30 weeks	Diosgenin-rich yam extract diopower 15 containing 8 mg diosgenin, 672 mg olive oil, glycerol fatty acid ester, vitamin E derivative and white beeswax (2 capsules/day = 50 mg), N.R = when doses are taken. Placebo (2 capsules/day) containing olive oil (672 mg), glycerol fatty acid ester, vitamin E derivative and white beeswax, N.R = when doses are taken, if placebo was matched in colour, smell and taste to active	12 weeks intake, with a 6-week washout period separating the crossover period	RBANS, MMSE-J (Japanese version)	Cognition: significant increase in RBANS between baseline and the 12-week endpoint intervention period (semantic fluency), total score independent of sex but age-dependent (significant for 47–81 years, sub-group analysis approached significance for ages 60–81) Retention/adherence: 3 participants withdrew from the study (31 originally), N.R = adherence Adverse events: none reported by participants

*AD* Alzheimer’s disease, *AE* Adverse event, *BOMC* Blessed Orientation Memory Concentration test, *CFT* Complex Figure Test, *COWA* Controlled Oral Word Association test, *CVLT* California Verbal Learning Test, *DAT* Divided Attention Task, *HAMD* Hamilton Rating Scale for Depression, *HVLT-R* Hopkins Verbal Learning Test-Revised, *MAC-Q* Memory Complaint Questionnaire, *MMSE* Mini-Mental State Examination, *MoCA* Montreal Cognitive Assessment, *N.R* Not reported, *POMS* Profile of Mood States, *RAVLT* Rey Auditory Verbal Learning Test, *RBANS* Repeatable Battery for the Assessment of Neuropsychological Status, *SCWT* Stroop Colour and Word Test, *SPMSQ* Short Portable Mental Status Questionnaire, *SRT* Selective Reminding Test, *TMT* Trail Making Test, *VFT* Verbal Fluency Test, *WAIS* Wechsler Adult Intelligence Scale, *WMS* Weschler Memory Scale

Across both populations, all studies were randomised, double-blind, placebo-controlled trials [20–40]. Twenty studies employed a parallel design [20–39], and one study utilised a cross-over design [40]. Three studies utilised the same intervention with two different doses (three comparison groups in total, including placebo) [20, 22, 28], and one study utilised two different interventions compared to a single control [29].

Eight studies were conducted in the USA [24, 25, 27–31, 36], three each in Australia [23, 32, 38] and Italy [26, 33, 39], two in India [21, 35] and one each in Korea [20], the Netherlands [22], the UK [34], China [37] and Japan [40]. Two studies were published from 1995 to 1998 [22, 33], ten published between 2000 and 2010 [21, 23–25, 27, 30–32, 35, 36], with the remaining nine between 2012 and 2020 [20, 26, 28, 29, 34, 37–40].

Fifteen studies reported methods of recruitment [20, 22–26, 28, 29, 32, 35–40], with twelve studies conducted in community settings (audio, visual, and print media, universities) [20, 23–25, 28, 29, 32, 35–38, 40] and three in clinical settings (general practice and outpatient clinics) [22, 26, 39].

### Participants

Across the included studies, the total sample size (at baseline) was *N* = 1891, with 19/21 studies reporting participant sex (*N* = 1798; 61% were female) [20–25, 28–40]. The mean age of participants reported across 20/21 studies was 65.43 years [20–34, 36–40], SD_pooled_ = 13.95 (for 17 studies) [20–23, 25, 27–33, 36–40]. Individual studies ranged from 28 [40] to 262 participants [31]. A total of 755 participants were from the nine SCI studies [20–22, 26, 28, 33, 35, 37, 38], compared to 1136 from the twelve studies in older adults without SCI [23–25, 27, 29–32, 34, 36, 39, 40].

### Eligibility criteria and global cognition measures

All twenty-one studies utilised cognitive scales or tests [20–22, 25–33, 35, 36, 38, 39], medical questionnaires [23, 37], self-reports of cognitive function [22, 24, 26, 27, 29–31, 34, 40] or clinical questionnaires [23, 24, 32, 38], to determine the eligibility for their respective study. Nineteen of these studies utilised a validated measure to test cognitive functioning [20–33, 35, 36, 38–40]. Fifteen out of twenty-one studies utilised the Mini-Mental State Exam (MMSE), as a measure of global cognition [20–22, 25–33, 35, 36, 38, 39]. The MMSE cut-off score for participant inclusion varied between studies and populations. For SCI studies, one utilised a cut-off of ≥ 20 [22], another a range of 20–27 [26], one with ≤ 24 [35], four studies with a cut-off of ≥ 24 [21, 28, 33, 38] and one of ≥ 25 [20]. For studies on older adults without SCI, one reported a cut-off of ≥ 23 [29], one with a range of 24–29 [25], three with a cut-off of ≥ 24 [27, 30, 32], one ≥ 26 [31] and one each > 26 [36] and ≥ 27 [39]. Overall, studies with an SCI population reported lower cut-off scores and ranges for participant inclusion.

Other scales included the Blessed Orientation Memory Scale (BOMC) [24], Weschler Memory Scale (WMS) [28, 29, 35], Clinical Dementia Rating (CDR) Scale [20], Memory Complaint Questionnaire (MAC-Q) [28] and Short Portable Mental Status Questionnaire (SPMSQ) [29]. Overall, four studies utilised a global cognition measure as their primary or secondary outcome measure, with three of these using the MMSE [26, 29, 39], and one the WMS (logical subset score of < 6) [35].

### Intervention and control type

Seventeen out of twenty-one studies used a herbal supplement [21, 23–26, 28–33, 35–40] with most studies utilising a form of *Ginkgo biloba* [23, 25, 29–31, 36–38] or *Bacopa monnieri* as a primary ingredient in their intervention [21, 24, 26, 32, 35, 39]. Two of these seventeen studies used a combination formula (one containing *Ginkgo biloba* and 45 other herbs, minerals and vitamins specifically made for women [38]) (the other containing *Bacopa monnieri*, lycopene, astaxanthin and vitamin B12 [39]). In addition, one study each used a spearmint extract (*Mentha spicata* L.) [28], a standardised ginseng extract (G115) [33] and a diosgenin-rich yam extract (diopower 15) containing vitamins, oils and beeswax [40], and one used a nutritional supplement (OPC Synergy® plus Catalyn) containing buckwheat, teas, and fruit and vegetable extracts, as a secondary intervention [29].

Two of the remaining four studies utilised a herbal combination via a liquid solution (*Ginkgo biloba*, alcohol/water solution [22] and SRM [*Salvia officinali*s L., *Rosmarinus officinalis* L. and *Melissa officinalis* L.]) [34], one study utilised a nutrient based juice (sweetened cranberry juice) [27], and one study used an oral capsule containing a type of fungus (*Tremella fuciformis*) [20]. Twenty out of twenty-one studies reported a method of administration [20–34, 36–40] with ten administering the intervention orally via capsule [20, 21, 25, 26, 28–30, 33, 37, 40], seven utilised tablets [23, 24, 31, 32, 36, 38, 39] and three a liquid solution [22, 27, 34].

In terms of the control groups, all studies stated that they utilised a form of placebo. Nineteen out of twenty-one studies detailed the type of placebo employed [20–28, 30–34, 36–40], with ten studies utilising a capsule [20, 21, 25, 26, 28, 30, 33, 36, 37, 40], six a tablet [23, 24, 31, 32, 38, 39] and three an oral liquid [22, 27, 34]. Eight out of twenty-one studies (38%) provided some information on the ingredients contained in the placebo [22, 28, 29, 34, 36–39]. Five of these studies used a form of placebo containing active herbal or nutritional ingredients [22, 29, 34, 38, 39], with the remaining three using a placebo containing inert substances [28, 36, 37]. Overall, 17/21 studies stated that they matched their placebo with the intervention [20–27, 30–33, 36–40]. Nineteen out of the twenty-one studies sufficiently detailed the dose of intervention [20–32, 34–36, 38–40], and seventeen detailed the dose of placebo [20–22, 24–28, 30, 31, 33, 34, 36–40].

The total duration of studies ranged from 2 weeks [34] to 9 months [33]. The majority of studies were 12 weeks [21, 23, 24, 32, 35, 37], 6 weeks [27, 30, 31, 36] or 8 weeks [20, 26, 39] in length. Four studies employed a washout, run-in or withdrawal period; two before the trial began [22, 39], one between two cognitive testing periods (midpoint-12 weeks and endpoint-24 weeks) [21] and one for 6 weeks separating the intervention cross-over period [40]. One study utilised a 6-week placebo intake period for the control group, with 12 weeks of intervention for the treatment group [24].

### Primary and secondary measures of cognition

Measures of cognition varied across the included studies. The most common were the Wechsler Adult Intelligence Scale (WAIS; *n* = 6) [21, 24, 27, 29, 31, 36], the Weschler Memory Scale (WMS; *n* = 6) [21, 27, 30, 31, 35, 36], the Rey Auditory Verbal Learning Test (RAVLT; *n* = 5) [21, 22, 24, 32, 39], the Stroop Colour and Word Test (SCWT; *n* = 4) [24, 27, 29, 30], the Trail Making Test (TMT; *n* = 4) [27, 29, 32, 39] and the Mini-Mental State Examination (MMSE; *n* = 4) [26, 29, 39, 40].

### Retention and adherence

Nineteen of the twenty-one studies consistently reported retention, with an average retention rate of 92% across the studies [20–26, 28, 30–38, 38–40]. Comparatively, only 7/21 studies specifically reported treatment adherence, with an overall average of 93% across both intervention and placebo usage [20, 22, 24, 26, 28, 33, 38, 38].

### Risk of bias within and across studies

**Fig. 2:**
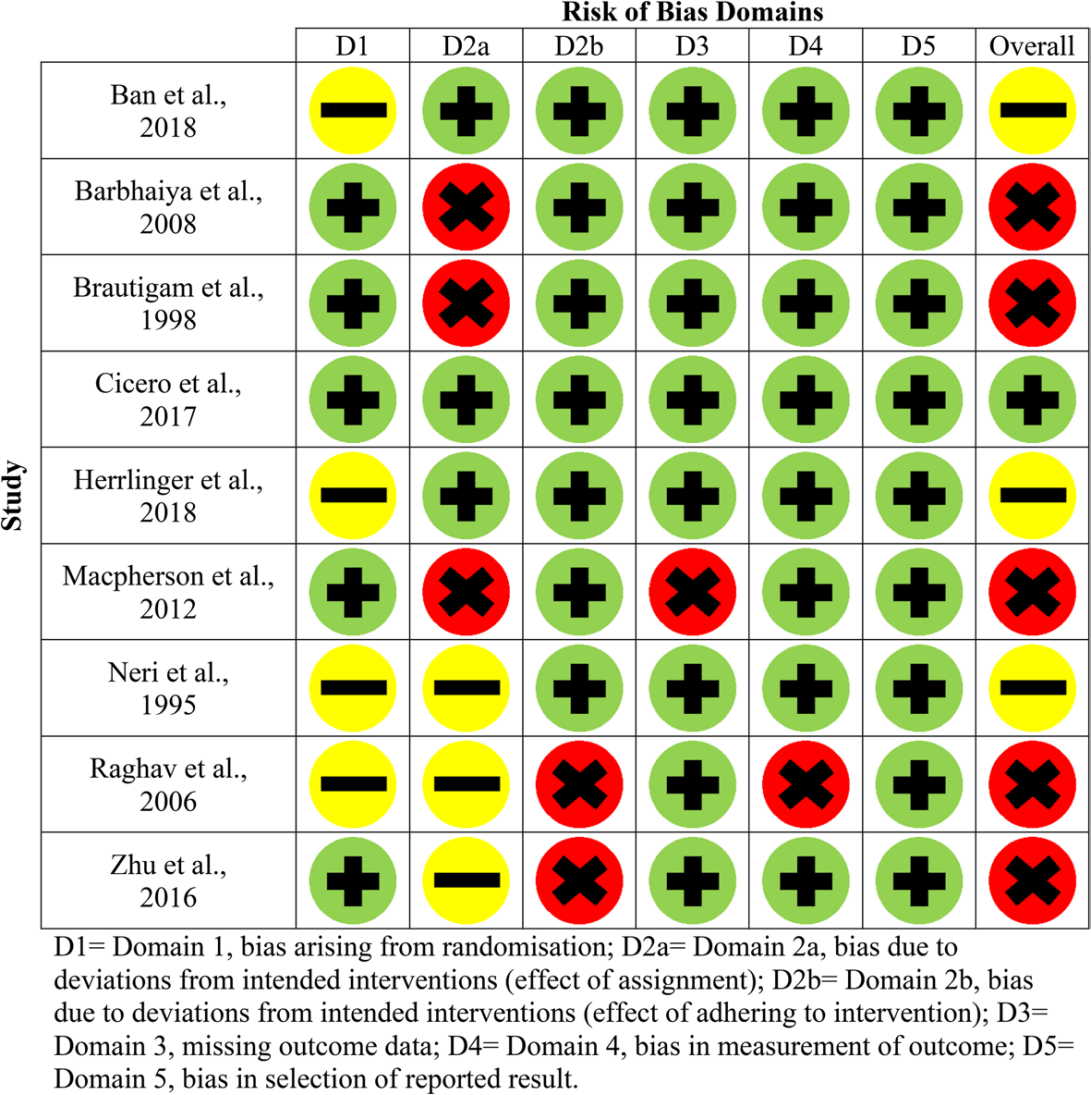
Risk of bias domains for older adults with SCI (parallel studies)

**Fig. 3:**
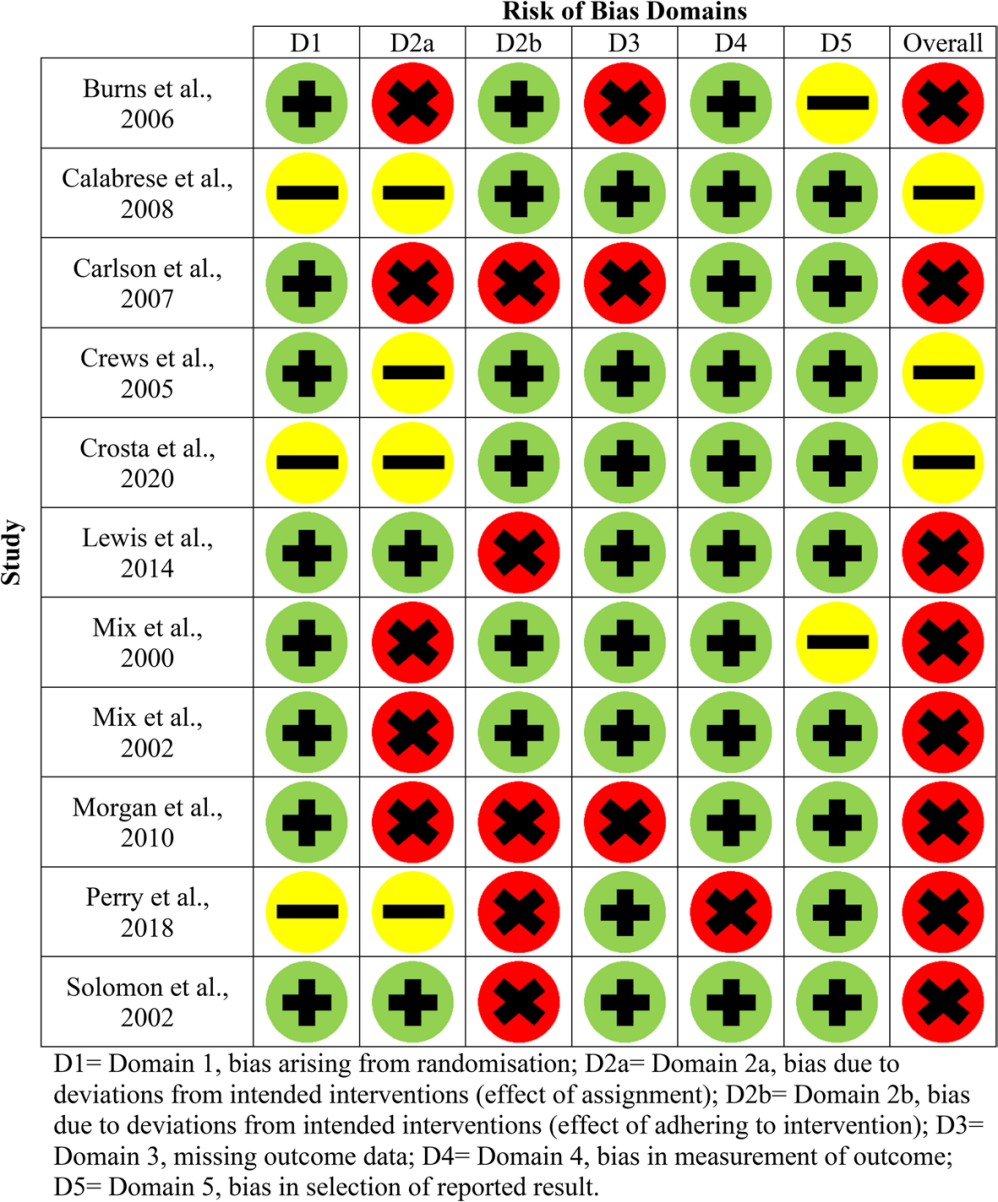
Risk of bias domains for older adults without SCI (parallel studies)

**Fig. 4:**
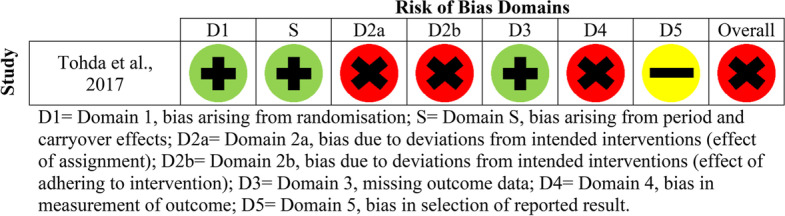
Risk of bias domains for older adults without SCI (cross-over study)

Figures 2 and 3 (parallel studies) and Fig. 4 (cross-over study) provide a summary of each of the risk of bias domains, and an overall risk of bias assessment, for each of the twenty-one included studies. Green circles indicate that the domain or study has been evaluated as low risk, yellow as having some concerns and red as high risk.

Each article was assessed in terms of randomisation, intended interventions (effect of assignment and effect of adhering to the intervention), missing outcome data, measurement of outcomes and selection in reported results. In terms of the overall risk of bias assessment, only one study was deemed as low risk [26], some concerns were found for 6/21 studies [20, 24, 27, 28, 33, 39] and the remaining fourteen were deemed to be high risk [21–23, 25, 29–32, 34–38, 40]. Between populations, three SCI studies were assessed as having some concerns [20, 28, 33], five were deemed high risk [21, 22, 35, 37, 38] and one was deemed low risk [26]. For the non-SCI studies, three were assessed as having some methodological concerns [24, 27, 39], and the remaining nine were deemed as high risk [23, 25, 29–32, 34, 36, 40].

Despite all twenty-one studies stating the method of intervention assignment was randomised, only fourteen sufficiently detailed the randomisation process and were deemed as low risk [21–23, 25–27, 29–32, 36–38, 40]. Intended interventions (effect of assignment to interventions) were adequately reported in five studies (low risk) [20, 26, 28, 29, 36], nine were assessed as high risk [21–23, 25, 30–32, 38, 40] and the remaining seven have some concerns [24, 27, 33–35, 37, 39]. Thirteen studies were assessed as low risk for reporting on intended interventions (effect of adhering to the intervention) [20, 22–24, 26–31, 33, 38, 39] and eight as high risk [24, 29, 32, 34–37, 40].

In terms of missing outcome data (domain three), 17/21 studies were evaluated as low risk [20–22, 24, 26–31, 33–37, 39, 40], and the remaining four were assessed as high risk [23, 25, 32, 38]. Within domain 4 (measurement of outcomes), 18/21 studies were deemed as low risk [20–33, 36–39], and the remaining three were as high risk [34, 35, 40]. Eighteen out of twenty-one studies were assessed as low risk for selection in reported results (domain 5) [20–22, 24–29, 31–39], with some concerns for only three studies [23, 30, 40]. In terms of bias arising from period or carryover effects (domain S) in the cross-over study, this was deemed as low risk [40].

### Study results

Results for all twenty-one studies are outlined below including intervention efficacy on cognitive function, adverse events and risk of bias.

#### Intervention efficacy in low risk of bias study

Only one study was deemed to be low risk for all domains, in terms of the overall methodological assessment. This 2017 randomised, double-blind placebo-controlled trial was conducted by Cicero and colleagues [26]. Participants were 30 older adults with self-perceived cognitive decline and ingested either a *Bacopa monnieri* formulation or a placebo capsule for 8 weeks; MMSE was measured at each time point. In terms of intervention efficacy, a significant increase in MMSE score was found from baseline to endpoint in the treatment arm. Furthermore, a significant increase in score was also found in the treatment group at the endpoint, compared to placebo, demonstrating a significant improvement in cognitive function for the intervention group across time and between groups [26]. Only one adverse event was reported an aftertaste from active product intake.

#### Intervention efficacy in remaining SCI studies

Across time, a significant improvement in at least one cognitive outcome for participants in the intervention group (compared to placebo) was found in 6/8 of the remaining SCI studies [20, 21, 28, 33, 35, 38]. Improvements were mostly found in the areas of memory (working, spatial, short-term, retention and logical) [20, 28, 33, 35, 38] and executive functioning [20, 21, 35]. Three of the eight studies utilised a capsule containing a herbal extract: one contained *Bacopa monnieri* [21], one spearmint extract (*Mentha spicata L*.) [28] and one standardised ginseng extract [33]. One study used a combination supplement (tablet), containing 46 herbs, vitamins and minerals (mainly consisting of *Ginkgo biloba*, *Silybum marianum* dry fruit (St. Mary’s thistle) and *Vitis vinifera* dry seed (grape seed)) [38]. An additional study utilised a capsule containing *Tremella fuciformis* (a type of fungus) [20], and the last study did not specify an administration method but used a standardised extract of *Bacopa monnieri* [35].

Of these six studies, mild to moderate adverse events were reported in three of them [28, 35, 38]. Knee pain, myalgia, headaches and heartburn were reported in the study conducted by Herrlinger and colleagues utilising *Mentha spicata* L. as their intervention [28]*.* These adverse events were reported for both the treatment and placebo groups; however, heartburn experienced by a participant in the 600 mg/day *Mentha spicata* L. group was deemed as ‘probably related’, compared to all other events deemed as ‘not related’ [28]. One participant withdrew due to maculopapular rashes in the intervention group (*Bacopa monnieri*), in the study conducted by Raghav and colleagues [35]. Two participants withdrew from the study conducted by Macpherson and colleagues using a combination formula containing *Ginkgo biloba* (intervention) [38]. One participant withdrew due to nausea and vomiting in the intervention group and one in the placebo group due to a mild rash [38].

In relation to the risk of methodological bias for all six SCI studies with an improvement in cognitive functioning, two of the studies utilising *Bacopa monnieri* [21, 35] and one using *Ginkgo biloba* (combination supplement) [38] were deemed as being high-risk. The remaining three were assessed as having some concerns in terms of methodological reporting [20, 28, 33].

For the two studies that did not find an improvement in cognitive functioning between groups (intervention *cf*. placebo) or across time, both reported adverse events with the use of *Ginkgo biloba* alcohol/water extract (drops) [22] and a herbal/dietary supplement also containing *Ginkgo biloba* [37]. Gastrointestinal upset was reported as the main adverse reaction for both studies [22, 37] with dizziness, headaches and sleep disturbance also reported in the *Ginkgo biloba* alcohol/water extract study [22]. In total, Brautigam and colleagues reported adverse events for 25 participants across both the placebo and intervention groups [22]. In terms of the second study, it is not known whether the two participants who reported adverse events were receiving the intervention or placebo [37]. Overall, both studies were deemed as high risk in terms of the methodological risk of bias assessment.

#### Intervention efficacy in non-SCI studies

Overall, 7/12 non-SCI studies reported a significant improvement in cognitive functioning in the intervention group (compared to placebo), across time [23, 24, 30–32, 39, 40]. The most common improvements were in memory (long-term storage, retrieval, delayed recall, recognition) [23, 24, 31, 32], executive functioning [24, 30, 39, 40] and language [30, 39, 40]. Two studies used a form of *Ginkgo biloba* capsule [23, 30]; one used a *Ginkgo biloba* tablet [31]; two used a form of *Bacopa monnieri* tablet [24, 32]; one used an antioxidant combination formula (tablet) containing *Bacopa monnieri*, lycopene, astaxanthin and vitamin B12 [39]; and one a diosgenin-rich yam extract capsule [40]. An additional study reported a significant improvement in executive functioning across time, using a combined herbal and nutritional supplement containing Ginkgo (Ginkgo Synergy® plus Choline) [29]. A significant improvement in verbal fluency was also found in the secondary intervention group (across time, compared to the Ginkgo and placebo groups) using OPC Synergy®, a dietary supplement (plus Catalyn) [29]. A further study using a *Ginkgo biloba*-based supplement found a significant improvement in a list learning strict task in the placebo group only, across time [25].

Seven of the nine total studies were deemed as having a high methodological risk of bias [23, 25, 29–32, 40], with the remaining two (using a form of *Bacopa monnieri*) having some concerns [24, 39]. Five of the nine studies reported adverse events, in both the placebo and intervention groups (Ginkgo or *Bacopa monnieri* interventions) [24, 25, 29, 31, 32]. An additional two studies reported adverse events only occurring in the intervention group using a *Ginkgo biloba* capsule [23] or an antioxidant combination formula containing *Bacopa monnieri* [39]. The most common events reported across 6/7 studies were gastrointestinal issues (including nausea, abdominal cramps, digestive problems) [24, 25, 31, 32] and sleep disturbance, with the use of *Ginkgo biloba* [23] or placebo [29]. Exacerbation of sinusitis (*n* = 1) and a serious but short-term event of hepatitis E (*n* = 1) were reported as non-treatment-related adverse events in the remaining study [39].

For the three remaining non-SCI studies, all reported no significant improvements across time in cognitive functioning (for both intervention and placebo groups), nor between groups [27, 34, 36]. One study used a Ginkgo tablet [36], one used a sweetened cranberry juice [27] and the other used a liquid solution of SRM *Salvia officinalis* L., *Rosmarinus officinalis* L. and *Melissa officinalis* L. [34].

Adverse events were monitored in two of the three studies; however, no events were reported in one (SRM solution) [34], and the other study did not report serious events or document mild/minor events (sweetened cranberry juice intervention) [27]. Two of the three studies were deemed as having a high methodological risk of bias [34, 36], and the remaining one had some concerns [27].

## Discussion

Overall, twenty-one studies were identified for inclusion in this review, nine with an SCI population [20–22, 26, 28, 33, 35, 37, 38] and twelve studies utilising older adults without SCI [23–25, 27, 29–32, 34, 36, 39]. Outcomes were mainly positive, with 14/21 studies overall reporting improvements in at least one area of cognitive functioning across time, in the intervention group (compared to placebo) [20, 21, 23, 24, 26, 28, 30–33, 35, 38–40]. Overall, only one study (using an SCI population) was assessed as having a low methodological risk of reporting, conduct and quality of trial design bias [26]. Due to the heterogeneous nature of eligible studies (including cognitive measures and interventions used and the type of data analysis and reporting conducted) and the large number of studies (14/21) with a high methodological risk of bias [21–23, 25, 29–32, 34–38, 40], a certainty of evidence analysis (GRADE) was not conducted.

### Extent of literature using herbal and nutritional medicines for older adults with and without SCI

Despite the growing interest in the prevention and treatment of cognitive decline in older adults [8, 9], review search outcomes were not reflective of this interest, given that most of the articles eligible for this review were conducted prior to 2018. Editorials, book chapters, reviews and opinion articles seem to be more common formats of evidence, compared to research using the ‘gold standard’ method, randomised control trials (RCTs) [41, 42] (Fig. 1).

Measurement of cognitive change was not common in the literature; rather, relevant population studies focussed on biochemistry or progression to MCI or dementia. This was an unexpected outcome during the records search. Cognitive testing and assessment are generally affordable and accessible methods of providing insights into an individual’s current cognitive functioning and any decline over time [43]. However, there are currently no recommendations for specific primary or secondary outcome measures of cognition to determine a clinically significant improvement in cognitive function [44]. It has recently been recommended that composite outcomes including the monitoring of dementia risk factors alongside changes in cognition may be advantageous in preclinical dementia [44].

For cognitive outcome measures, an effect size of 0.40 has been reported as a clinically meaningful improvement for cognitive training interventions in healthy older adults and those with MCI or dementia [45]. This could be applied in studies utilising cognitive outcomes in people with SCI to determine whether a change in cognitive function is clinically meaningful, particularly in light of potential ceiling effects in this relatively unimpaired group. Future research should strive to investigate appropriate cognitive measures to detect a clinically significant change in SCI and implement gold standard, high-quality research methods to produce informative and translational outcomes.

### Study characteristics

In terms of participant characteristics, across the twenty-one studies, there were more females (61%) compared to male participants. It is difficult to ascertain the true difference in the prevalence of SCI between the sexes, as a larger number of females (rather than males) are participating in these studies. Furthermore, inconsistencies in reporting prevalence between the sexes are typical in this field, again making it hard to determine whether SCI affects more females or males [2]. However, research within the area of cognitive decline suggests that females have a greater cognitive reserve but have a faster rate of cognitive decline (particularly, in the areas of global cognition and executive function) compared to males [46]. This outcome has been confirmed in dementia research. Dementia is reported to be the leading cause of death in women, with twice as many females compared to males being affected by the disease [47]. Further SCI prevalence research needs to be conducted to determine the true prevalence of SCI, between the sexes.

A high number of older adults reporting SCI are within the 60–64 year age range [2], compared to studies included in this review that saw an overall average age of 65 years for participants. Sex and age outcomes derived from this review highlight the importance of finding a way to address low research participation in males and monitoring the faster rate of female decline.

In terms of participant retention and adherence to treatment, these were both surprisingly high across the studies at an average of 92% and 93%, respectively, despite the literature suggesting these figures are quite difficult to achieve [48]. These outcomes should be considered with caution due to the subpar methodologies used to treat missing values.

### Study methodologies

Reflective on previous research, the eligibility criteria for participation across the SCI and non-SCI studies were inconsistent [8, 9], with varying scales, tests and questionnaires used, particularly for the MMSE [20–22, 25–33, 35, 36, 38, 39]. Research investigating the diagnostic accuracy (sensitivity, specificity, positive and negative predictive power) of MMSE cut-off scores in detecting cognitive dysfunction found that scores of ≤ 26 showed optimal sensitivity and specificity balance, with a correct classification of MCI and dementia in older adults to be 90% [49]. The varying MMSE cut-offs used here (≥ 20, > 25 and ≤ 24) may have incorrectly classified participants (with and without SCI), potentially impacting the study outcomes. This interpretation is further supported by the identification of all eligible studies in this review not using a combination of self-report cognitive concerns (in line with the definition of SCI), cognitive scales (such as the MMSE), a general health questionnaire (including non-diagnosis of MCI or dementia) and screening of mental health conditions. Reliability on only one or two measures for classification of an impairment (or no impairment) is problematic and certainly requires future attention within this area of clinical practice and research.

An additional concern regarding the methodologies of accepted studies in this review is the large number of those deemed as having a high risk of bias, particularly within the bias due to assignment and adherence domains [21–23, 25, 29–32, 34–38, 40]. Either intention-to-treat (ITT) or modified intention-to-treat (mITT) approaches were not employed for participants with missing outcomes or outlier data, with participants being excluded completely from the analysis despite being randomised. Future studies in this field should consider using appropriate analysis to treat missing or outlier data, for post-randomisation outcomes as detailed above. The blinding of participants and other individuals involved in the trial was also identified as a concern. However, it is difficult to ascertain whether it was in fact the blinding process itself that was not conducted appropriately in these studies or if it was simply not reported sufficiently according to the ROB assessment standards. Future studies should look to adopting greater transparency and accuracy in the process (specifically stating who was blinded and how), as this would go a long way in demonstrating non-biassed outcomes.

### Intervention efficacy on cognitive function and safety

Despite the majority of studies reporting positive results [20, 21, 23, 24, 26, 28, 30–33, 35, 38–40], a large number of these were deemed as having an overall high methodological risk of bias [21, 23, 30–32, 35, 38, 40]. These methodological concerns unfortunately do not assist in determining the true efficacy of herbal and nutritional medicines on cognitive functioning for older adults with and without SCI. This is particularly the case for *Ginkgo biloba* [23, 25, 29–31, 36–38] and *Bacopa monnieri* [21, 24, 26, 32, 35, 39], given how common they were as interventions across the accepted studies of this review.

The efficacy of *Ginkgo biloba* has been consistently unclear across the spectrum of cognitive decline. An earlier review investigating RCTs using *Ginkgo biloba* for the treatment of dementia [50] highlighted the concerns around the low quality of studies available, namely to do with utilisation of unsatisfactory methods. However, on a positive note, adverse effects found with the use of *Ginkgo biloba* (across the accepted studies in this review) appeared to be consistent with those reported with the use of a placebo [23, 29, 31, 38], indicating that *Ginkgo biloba* may be comparable in terms of safety with placebo intake. These results are in line with what has been found previously in a dementia population [50].

### Strengths and limitations of the review

This review had several strengths. A broad and extensive literature search was conducted (in accordance with the aims and PICOS criteria of the review), comprehensively summarising the overall current state of the field. The lack of high-quality research has been addressed, highlighting the specific aspects which require improvement in future studies. The concerns surrounding the classification of SCI and the disparities between current research outcomes and clinical statistics have been presented.

There were a number of limitations to this review. First, the establishment of article alerts from 2018 until the completion of the review meant that despite the authors’ best efforts to monitor the addition of newly published research in each database, it is acknowledged that alerts may not have been the most appropriate way to capture all potential studies for inclusion. Furthermore, a meta-analysis was not feasible due to the inconsistent classification of SCI and non-SCI samples and the varying cognitive testing measures. The infancy of this area of research (despite broad interest from the general public) makes it difficult to conduct such an analysis at this time. The population was difficult to define due to the inherent heterogeneity of definitions and lack of consensus within the field, particularly with reference to the age range selected (despite being guided by the US CDC’s definition), and the terminology and language used (e.g. person-centred terminology). It is also acknowledged that the current review did not take into consideration the varying terminology utilised to classify ‘older adults’. The exclusion of non-English language studies, the initial article screening conducted by one reviewer and the search strategy developed in consultation with only one librarian were further limitations.

### Recommendations for future research

First and foremost, an increased understanding and awareness of the features and characteristics of SCI needs to occur [8, 9]. This should be considered in collaboration with the difference between the presentation of older adults without SCI and those with MCI, in line with the cognitive decline continuum [2]. Future research should aim at clarifying the characteristics, classification measures and features of SCI to allow for more homogeneous sample classification. Overall, by better understanding of SCI, this may provide greater support for outcomes in high-quality efficacy studies utilising herbal and nutritional medicines as a means of managing self-perceived (or subtle) cognitive decline and, ideally, lowering dementia risk or facilitating the secondary prevention of dementia.

The development of a standardised outcome measure package (including cognitive testing, medical questionnaires, self-reports and mental health questionnaires) for use in SCI clinical trials would be the next step in moving the field forward. Increased accuracy in the differentiation between healthy older adults (without SCI) and those with SCI would assist in determining whether herbal and nutritional medicines have a positive effect on cognitive outcomes for this population.

### Conclusions

Whilst most studies deemed eligible for inclusion in the review found positive results (particularly, those that used *Ginkgo biloba* or *Bacopa monnieri*), these outcomes need to be considered with caution, due to the high risk of methodological bias found. The literature in this area is in its infancy, with concerns around population and intervention heterogeneity evident. The use of supplements for cognition by older people is an area that attracts much interest from the community, yet our review shows that high-quality research on efficacy and safety is somewhat lagging.

This review has provided an insight into the current state and quality of the literature on the safety and efficacy of cognitive function of herbal and nutritional medicines in older adults with and without SCI.

## Data Availability

Not applicable.

## Footnotes

1 Older adults (with and without SCI) were defined as aged 45 years and older, in accordance with the US Centers for Disease Control and Prevention (CDC) population-based statistics on Subjective Cognitive Decline and Aging [7].
